# Targeting tumor-associated macrophages with STING agonism improves the antitumor efficacy of osimertinib in a mouse model of *EGFR-*mutant lung cancer

**DOI:** 10.3389/fimmu.2023.1077203

**Published:** 2023-02-16

**Authors:** Ziying Lin, Qiwei Wang, Tao Jiang, Weihua Wang, Jean J. Zhao

**Affiliations:** ^1^ Department of Cancer Biology, Dana-Farber Cancer Institute, Boston, MA, United States; ^2^ Department of Pulmonary and Critical Care Medicine, The First Affiliated Hospital of Sun Yat-sen University, Guangzhou, Guangdong, China; ^3^ Department of Biological Chemistry and Molecular Pharmacology, Harvard Medical School, Boston, MA, United States; ^4^ Broad Institute of Harvard and MIT, Cambridge, MA, United States; ^5^ Laboratory of Systems Pharmacology, Harvard Medical School, Boston, MA, United States

**Keywords:** lung cancer, EGFR-mutant, Osimertinib, STING agonist, tumor-associated macrophages

## Abstract

**Introduction:**

Despite the impressive clinical response rate of osimertinib, a third-generation EGFR-TKI, as a frontline treatment for patients with EGFR-mutant non-small-cell lung cancer (NSCLC) or as a salvage therapy for patients with T790M mutation, resistance to osimertinib is common in the clinic. The mechanisms underlying osimertinib resistance are heterogenous. While genetic mutations within EGFR or other cancer driver pathways mediated mechanisms are well-documented, the role of tumor cell and tumor immune microenvironment in mediating the response to osimertinib remains elusive.

**Methods and results:**

Here, using a syngeneic mouse model of EGFR-mutant lung cancer, we show that tumor regression elicited by osimertinib requires activation of CD8+ T cells. However, tumor-associated macrophages (TAMs) accumulated in advanced tumors inhibit CD8+ T cell activation and diminish the response to osimertinib. These results are corroborated by analyses of clinical data. Notably, reprogramming TAMs with a systemic STING agonist MSA-2 reinvigorates antitumor immunity and leads to durable tumor regression in mice when combined with osimertinib.

**Discussion:**

Our results reveal a new mechanism of EGFR-TKI resistance and suggest a new therapeutic strategy for the treatment of EGFR-mutant tumors.

## Introduction

Non-small cell lung cancer (NSCLC) remains one of the most prevalent malignant disease with high mortality (1). Epidermal growth factor receptor (EGFR) mutations are common in NSCLC, with the incidence being up to 15% among Caucasian patients and 50% among Asian patients (2, 3). EGFR tyrosine kinase inhibitors (EGFR-TKIs) have revolutionized the treatment landscape of NSCLC with *EGFR* mutations. Notably, osimertinib (AZD9291), a third-generation EGFR-TKI, shows superior therapeutic efficacy to earlier-generation EGFR-TKIs in patients with EGFR-TKI sensitizing mutations [exon 19 deletion (19del) or L858R mutation] (4). Despite potent anti-tumor activity, osimertinib rarely cures disease, as patients invariably acquire resistance to this drug. Resistance mechanism to osimertinib is highly heterogeneous, encompassing EGFR-dependent mechanism like the acquisition of resistance mutations, and EGFR-independent mechanisms like MET amplification, RAS–MAPK pathway activation, oncogenic fusions, and histologic transformation (5). However, our current knowledge fails to explain the resistance in 40-50% and 30-40% of cases with osimertinib as first or second-line line treatment (5),and our understanding of the mechanism that limits the efficacy of TKIs is still evolving.

Gurule et al. recently showed that EGFR-TKIs elicits interferon response in *EGFR*-mutant NSCLC, which is associated with improved therapeutic outcome (6), suggesting immune activation is critical for the therapeutic efficacy of EGFR-TKIs. However, how the interactions between tumor cells and tumor immune microenvironment affect TKI efficacy remains elusive. As immune activation contributes to effectiveness of many anti-cancer therapies, an immune suppressive microenvironment induced by tumor cells can render therapeutic resistance. For example, macrophage as an immune suppressive component has been reported to block T cell-mediated response and compromise the therapeutic outcome of chemotherapy and radiotherapy (7–9). Whether immune suppression also plays a role in restricting TKI efficacy is yet to be evaluated. Of note, previous studies showed that complete response (CR) could be achieved in early-stage lung cancer with EGFR-TKIs as neoadjuvant treatment (10), whereas EGFR-TKIs rarely induced CR among patients with advanced NSCLC (11–13), for which a highly immune suppressive microenvironment was established (14). We reasoned that tumor microenvironment may play a role in development of resistance to EGFR-TKIs.

Here, using preclinical mouse models and clinical datasets, we showed that T cell activation is important for the response of *EGFR*-mutant lung cancers to osimertinib. Notably, we found that immunosuppressive TAMs, which are enriched in more advanced tumors, diminished T cell activation upon osimertinib treatment, thus limiting the efficacy of osimertinib. Treatment with a systemically delivered STING agonist MSA-2 reprogramed TAMs and restored antitumor activity of osimertinib in eliciting T cell activation and durable tumor regression. Our findings highlight a new combination strategy to improve the therapeutic response of *EGFR* mutant-driven lung cancers to osimertinib.

## Methods

### Mice

All animal experiments described in this study were performed according to the animal protocols approved by the DFCI Institutional Animal Care and Use Committee (IACUC). Adenovirus expressing Cre recombinase (Ad-Cre) was intranasally injected into 8-week-old FVB mice carrying homozygously floxed alleles of *Trp53* (*Trp53^L/L^*). Alveolar epithelial (AE) cells were harvested from *Trp53^L/L^* mice one week after Ad-Cre administration, and were transduced with lentivirus carrying human *EGFR* exon 19del/T790M mutation. The resulting cells, which express Exon 19del/T790M EGFR with loss of expression of *Trp53*, are referred as PE. PE cells were transplanted to syngeneic FVB mice for tumor generation.

### Cell culture

Cells were cultured in a humidified incubator under 5% CO_2_ at 37°C. Tumor cells isolated from PE tumors were cultured in PDX medium [Ham’s F-12 and DMEM (Gibco) supplemented with 0.6% FBS (Gibco), 1 µg/mL Hydrocortisone (Sigma), 10 μg/mL Insulin (Thermo Fisher), 1 ng/mL Cholera Toxin (Sigma), 20 ng/mL EGF (Sigma), 100 μg/mL penicillin–streptomycin (Gibco)].

### Western blotting

Whole cell lysates were prepared using ice-cold RIPA buffer supplemented with protease and phosphatase inhibitor cocktail (Thermo Fisher). Equal amount of proteins were separated by 10% SDS-PAGE gel, and were transferred to polyvinylidene fluoride (PVDF) membranes. After blocking with 5% non-fat milk (Bio-Rad) in TBS plus 0.05% Tween 20 at room temperature, the membrane was incubated with in primary antibody overnight at 4°C. Blots were then incubated with fluorescently-labeled anti-mouse IgG (Rockland Immunochemicals, # RL610-145-002) or anti-rabbit IgG (Molecular Probes, # A-21109) at room temperature for 1 hour. Western blots were then scanned using Odyssey scanner (LI-COR).

### Cell viability assay

Tumor cells were seeded in 96-well plates at a density of 1000 cells per well and allowed to adhere overnight. Cells were then treated with serial dilutions of drugs for 3 days. Cell viability was assessed by Cell Titer Glo (Promega) according to manufacturer’s instructions. IC_50_ were generated using a non-linear regression model in GraphPad Prism 9.

### Tumor growth and treatment

1×10^6^ PE cells were resuspended in 100 μL serum-free DMEM containing 40% matrigel (Corning) and subcutaneously injected into 6 to 8-week-old FVB/N mice. Tumor growth was monitored by measuring the tumor size with a digital caliper. To monitor tumor growth, greatest longitudinal diameter (length) and the greatest transverse diameter (width) were measured with a digital caliper. Tumor volume was calculated by using a modified ellipsoid formula (0.52 × length × width^2^). Mice were euthanized by CO_2_ inhalation when tumor size met humane endpoints described in the IACUC protocols (20 mm diameter) or upon severe health deterioration.

For *in vivo* treatment, osimertinib was administered by oral gavage at a dosage of 10 mg/kg/day. MSA-2 was administered by intraperitoneal (i.p.) injection at a dosage of 20 mg/kg body weight every three days. To deplete CD8^+^ T cells, an anti-mouse CD8α neutralizing antibody (400 μg/mouse; clone YTS 169.4, BioXcell) was administered *via* i.p. every 3 days, starting from 48 hours before other treatments. For macrophage depletion, an anti-mouse CSF1R neutralizing antibody (clone AFS98, BioXcell) was dosed at 40 mg/kg *via* i.p. thrice a week, starting from 48 hours ahead of osimertinib treatment.

### Co-culture experiments

For *in vitro* co-culture experiments, TAMs (7-AAD^^−^^CD45^+^CD11b^+^F4/80^+^) were isolated from advanced PE tumors using a FACSAria II cell sorter (BD Biosciences). and cultured in DMEM medium supplemented with 10% FBS and 10 ng/mL mouse M-CSF (BioLegend, # 576404). 1x10^5^/well TAMs were seeded onto a 48-well plate and allowed to adhere overnight. Bone marrow-derived macrophages (BMDMs) were obtained from FVB/N mice by modifying previously described protocols (15, 16). Bone marrow cells were seeded on ultra-low attachment plates (Corning) or petri dishes (Falcon) and cultured in DMEM growth medium (DMEM + 10% FBS + 100 μg/mL penicillin–streptomycin) supplemented with 10 ng/mL mouse M-CSF (BioLegend, # 576404). On day 3, cell culture was top-up with fresh DMEM growth medium (same as original volume) with 10 ng/mL M-CSF. Cells were then incubated for another 4 days before harvesting adherent cells (BMDMs). Mouse CD8^+^ T cells were isolated from spleens of FVB mice using a mouse CD8^+^ T cell isolation kit (StemCell, # 19853). CD8^+^ T cells were cultured alone or co-cultured with TAMs or BMDMs at a ratio of 1:1 in RPMI 1640 supplemented with 10% FBS, 10 ng/mL mouse M-CSF, 0.055 mM 2-mercaptoethanol, 2 ng/mL IL-2 (Peprotech), 2.5 ng/mL IL-7 (Peprotech) and 50 ng/mL IL-15 (Peprotech) for 2 days.

### Tissue dissociation and flow cytometry

To obtain single-cell suspensions for flow cytometry, tumors were excised, minced and dissociated in collagenase/hyaluronidase buffer [DMEM with 5% FBS, 10 mM HEPES (Gibco), 100 μg/mL penicillin–streptomycin, 20 μg/mL DNase I (StemCell) and 1× collagenase/hyaluronidase (StemCell)] for 45 min at 37°C with agitation. Tumor-draining lymph nodes were isolated from tumor-bearing mice and were mashed through 70 μm strainer using plunger of a syringe. Red blood cells (RBC) were lysed using RBC lysis buffer (Life Technologies, # 00-4333-57).

For flow cytometry, cells were washed with cold FACS buffer (PBS containing 0.2% BSA and 5 mM EDTA). Cells were then stained with LIVE/DEAD Fixable Aqua Dead Cell Stain (Thermo Fisher), followed by blocking with anti-mouse CD16/32 (93, BioLegend) on ice. Next, cells were incubated in FACS buffer for 30 min on ice with antibodies specific to CD45 (30-F11, BioLegend), CD3 (145-2C11, BioLegend), CD8 (53-6.7, BioLegend), CD4 (RM4-5, BioLegend), TNF-α (MP6-XT22, BioLegend), IFN-γ (XMG1.2, BioLegend), Granzyme B (NGZB, eBioscience), CD44 (IM7, BioLegend), CD62L (MEL-14, BioLegend), Ki-67 (16A8, BioLegend), CD11b (M1/70, BioLegend), CD11c (N418, BioLegend), F4/80 (BM8, BioLegend), MHC II (M5/114.15.2, BioLegend), CD206 (C068C2, BioLegend), iNOS (CXNFT, eBioscience), TGF-β1 (TW7-16B4, BioLegend), Arginase 1 (IC5868A, R&D Systems), PD-L1 (10F.9G2, BioLegend).

For cytokine analysis, cells were stimulated with Leukocyte Activation Cocktail (BD Biosciences, # 550583) in RPMI medium (10% FBS) at the manufacture’s recommended concentration for 4 hours at 37°C/5% CO_2_. For intracellular staining, cells were fixed and permeabilized with Foxp3/Transcription Factor Staining Buffer Set (eBioscience, # 00-5523-00) before antibody incubation.

### Patient data analyses

Whole exome RNA-seq data of tumor tissues biopsied from eight patients with *EGFR*-mutant lung cancers were download from Gene Expression Omnibus (GEO) repository (https://identifiers.org/geo:GSE165019). Clinical information of these patients was obtained from a published study (6). Briefly, all eight patients were diagnosed at advanced stages (Stage IIIB or IV) and received an EGFR-TKI (osimertinib or erlotinib) as first-line treatment. Tumors were biopsied before treatment, and re-biopsied after ~2 weeks (10 days to 2 months) of treatment. The time to progression (TTP) ranged from 6.2 – 16.3 months, and the median and mean of the TTP were 10.8 and 10.9 months, respectively. Patients were grouped according to the duration of response [TTP of 0 – 8.6 month (n = 4 patients) or > 12 months (n = 4 patients)] (6).

The other two clinical datasets of NSCLC patients received surgical treatments were also analyzed. Data of Nat Genet 2020 cohort (17) were obtained from cBioPortal (http://www.cbioportal.org). GSE31210 (18) data were obtained from GEO database (https://www.ncbi.nlm.nih.gov/geo/).

Gene set enrichment analysis (GSEA) (19) was performed to compare the enrichment of gene signatures between two groups. Genes were first ranked according to log2 (fold change), using DESeq2 package in R software environment (20), and then analyzed using GSEAPreranked tool with the ‘classic’ method. Enrichment scores of TAM signature (21) and T cell inflamed signature (22) were generated by gene set enrichment analysis (ssGSEA), using GSVA R package.

Clinical data of EGFR-mutated advanced NSCLC treated with EGFR-TKIs as front-line treatment were also collected from The First Affiliated Hospital of Sun Yat-sen University. Inclusion criteria included: non-resectable NSCLC with EGFR driver mutations; received EGFR-TKI monotherapy as first-line treatment; follow-up information regarding the clinical response at 8th month of treatment was available. Eligible patients had pathologically confirmed clinical stage IV NSCLC (T1-4N0-2M1-2) and also underwent a core needle biopsy for mutation testing pretreatment. Clinical response was assessed at the 8th month of TKI treatment according to Response Evaluation Criteria in Solid Tumors (RECIST) version 1.1 (23). As all the included cases had developed distant metastasis, we carried out the analysis by only focus on the extent of primarily tumor, which was determined by T stage according to the 8^th^ Edition of the AJCC TNM Staging System (24).

### Statistical analyses

Statistical analyses were performed using GraphPad Prism v8 or SPSS software v26. Unpaired two-tailed *t* test (for normally distributed data) or Wilcoxon rank-sum test (for non-normal data) was used to compare two groups. For the comparisons of three or more groups, One-way ANOVA with Tukey’s multiple comparisons test (for normally distributed data) and Kruskal-Wallis nonparametric test (for non-normal data) were applied. Chi-squared test was used for categorical groups (therapeutic response, tumor staging). *P* value less than 0.05 was considered statistically significant.

## Results

### Osimertinib-elicited T cell activation is important for therapeutic efficacy of osimertinib *in vivo*

**Figure 1:**
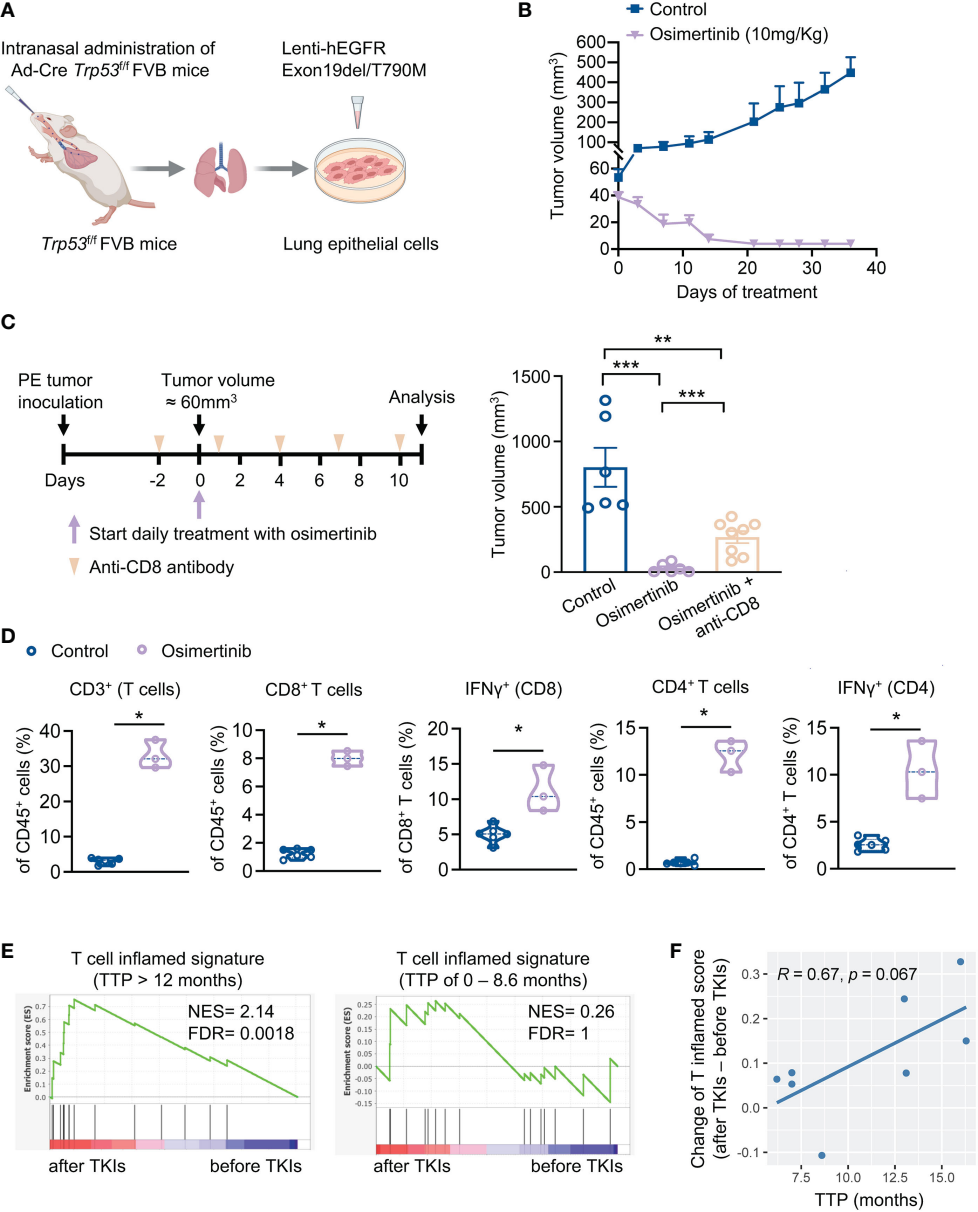
Osimertinib-elicited activation of T cells is important for the anti-tumor efficacy of osimertinib *in vivo.* **(A)** Generation of a syngeneic GEMM of *EGFR*-mutant tumors driven by Exon19del/T790M *EGFR* and loss of *Trp53* (referred as PE). **(B)** PE tumor growth in FVB mice treated with or without osimertinib. Treatment was started when tumor volume reached approximately 60 mm^3^. Control, n = 4; osimertinib (10 mg/kg, p.o., q.d.), n = 6. **(C)** PE tumor-bearing FVB mice were treated with osimertinib (10 mg/kg, p.o., q.d.) with or without an anti-CD8 neutralizing antibody. **Left**, schedule of tumor inoculation and treatments. **Right**, tumor volumes were measured after 11 days of treatment. Control, n = 6; osimertinib, n = 6; osimertinib + anti-CD8, n = 8. **(D)** Flow cytometry analysis of PE tumors in FVB mice after 11 days of osimertinib (10mg/kg) treatment. Treatment was started when tumor volume reached approximately 60 mm^3^. Each dot represents data from a single tumor. **(E, F)** Analysis of a cohort of eight patients with *EGFR*-mutant lung cancers who received an EGFR-TKI (osimertinib or erlotinib) as the first-line treatment. **(E)** Analysis of T cell inflamed signature in matched pairs of lung tumor biopsy collected before and after treatments. **Left**, patients with prolonged response [time to progression (TTP) > 12 months], n = 4 patients. **Right**, patients with short duration of response (TTP of 0 – 8.6 month), n = 4 patients. **(F)** Pearson correlation analysis of TTP with changes of enrichment scores of T cell inflamed signature upon EGFR-TKI treatments. Each dot represents one patient. n = 8 patients. Data are presented as mean ± SEM **(B, C)** or median with quartiles (violin plots, **D**). Two-tailed unpaired *t* test **(C, D)**. *P < 0.05, **P < 0.01, ***P < 0.001.

To investigate the role of immune response in the antitumor activity of EGFR-TKI, we developed a syngeneic genetically engineered mouse (GEM) model of lung cancer driven by loss of *Trp53* and expression of exon 19del/T790M *EGFR* (designated as PE) (**Figure 1A**). Primary tumors cells derived from PE tumors demonstrated constitutive activation of EFGR/ERK signaling pathway (**Supplementary Figure 1A**). As expected, osimertinib, but not erlotinib, inhibited EFGR/ERK pathway and growth of PE cells *in vitro* (**Supplementary Figures 1A, B**). We proceeded to evaluate the response of PE tumors to osimertinib *in vivo*. Because primary PE tumor cells could not be engrafted into the lungs of FVB mice *via* tail vein injection, we took an alternative approach by implanting PE cells subcutaneously in FVB mice for our study. Our initial treatment with osimertinib was started when PE tumors were relatively small (< 60 mm^3^). As shown in **Figure 1B**, osimertinib dosed at 10 mg/kg (p.o., q.d.) resulted in tumor regression, indicating that small early-stage PE tumors are highly sensitive to osimertinib. To determine whether the immune system plays a role in the antitumor activity of osimertinib, we depleted CD8^+^ T cells using an anti-CD8 antibody (**Supplementary Figure 1C**). Notably, therapeutic effect of osimertinib was significantly mitigated by CD8^+^ T cell depletion (**Figure 1C**). Consistently, we found that osimertinib treatment induced the recruitment of CD8^+^ and CD4^+^ T cells, which was accompanied by an increase of IFNγ-positive CD8^+^ and CD4^+^ T cells (**Figure 1D**, **Supplementary Figures 1D, E**), suggesting immunomodulatory properties of osimertinib.

To corroborate the relevance of T cell activation in the therapeutic efficacy of EGFR-TKIs, we re-analyzed a clinical cohort of 8 patients with *EGFR* mutant lung cancers (6). These patients received erlotinib or osimertinib as the first-line therapy and had tumor biopsies before and after the treatment. Analysis of tumor RNA-seq data showed that T cell inflamed signature was significantly enriched upon EGFR-TKI treatment in patients with prolonged response (TTP > 12 months) but not in patients with short duration of response (TTP of 0 – 8.6 month) (**Figure 1E**). Moreover, the increase of T cell inflamed score upon EGFR-TKI treatment and longer TTP exhibited a correlation with a trend toward significance (*p = 0.067*, **Figure 1F**). Together, our findings highlight the importance of adaptive immune activation in the anti-tumor activity of EGFR-TKIs.

### Advanced PE tumors dominated by M2-like tumor-associated macrophages (TAMs) are resistant to osimertinib

**Figure 2:**
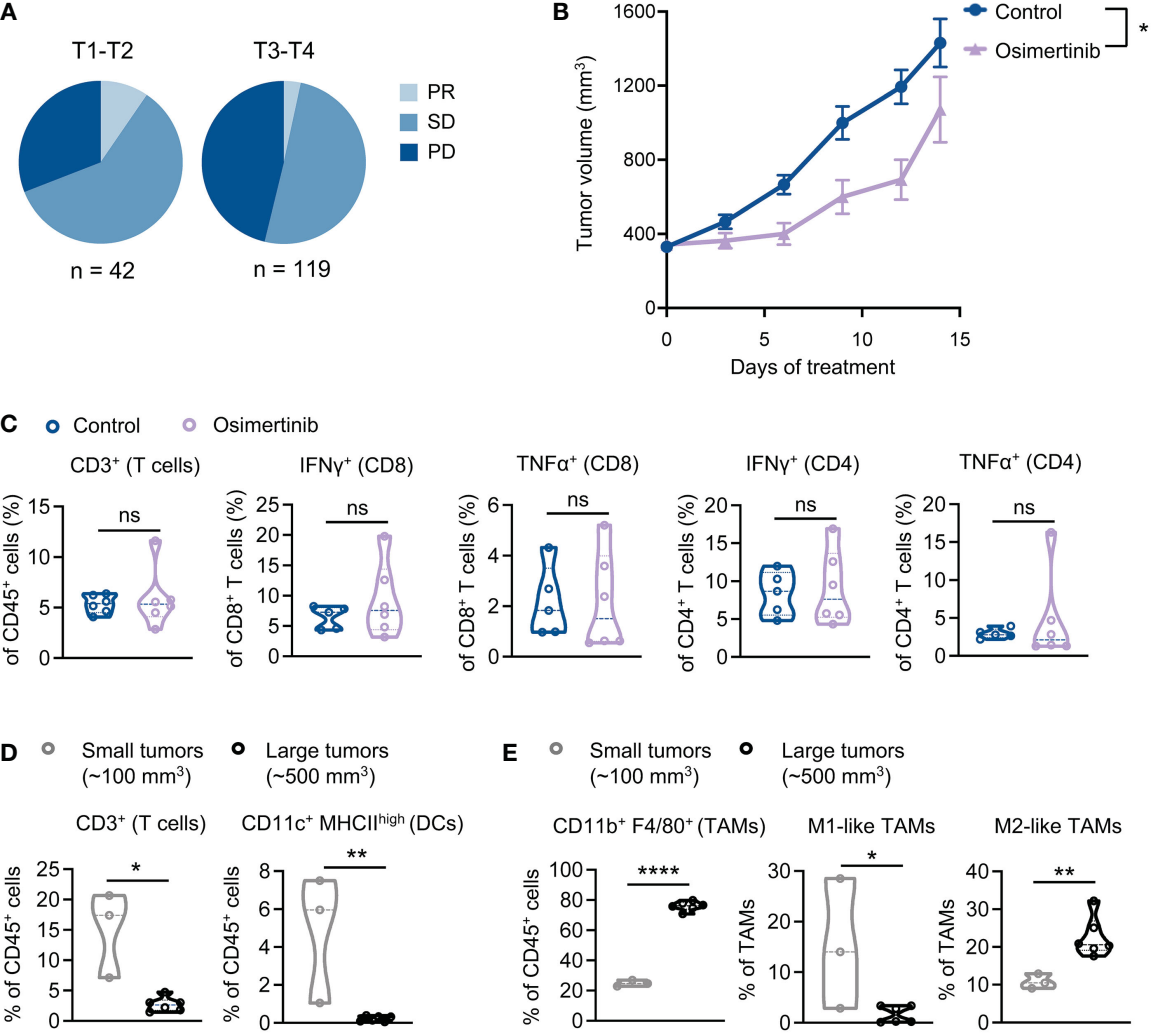
Advanced PE tumors dominated by M2-like tumor-associated macrophages (TAMs) are resistant to osimertinib. **(A)** Clinical response to EGFR-TKI as a front-line therapy in patients with *EGFR*-mutant advanced NSCLC. Response assessment was performed at the 8^th^ month of TKI treatment. PR, partial response; SD, stable disease; PD, progressed disease. Tumors were classified as T1-T2 and T3-T4 according to tumor staging in TNM staging system. Difference in clinical response was assessed by Chi-square test (χ^2 =^ 7.4, P=0.025). **(B, C)** PE tumor-bearing FVB mice were subjected to osimertinib (10 mg/kg, p.o., q.d.) when tumors reaching 400 mm^3^. **(B)** PE tumor growth. Control, n = 10; osimertinib, n = 10. **(C)** Flow cytometry analysis of PE tumors after 2 weeks of osimertinib treatment. Each dot represents data from a single tumor. **(D, E)** Flow cytometry analysis comparing the immune profiles of treatment naïve small (~100 mm^3^) and large (~500 mm^3^) PE tumors. Each dot represents data from a single tumor. **(D)** Comparing small and large PE tumors for T cells and dendritic cells (DCs) infiltration. **(E)** Comparing small and large PE tumors for TAMs. TAMs were further analyzed to identify M1-like (MHC-II^high^ CD206^−^) and M2-like (MHC-II^low^ CD206^+^) polarization phenotypes. Data are presented as mean ± SEM **(B)** or median with quartiles (violin plots, **C**–**E**). Two-way ANOVA **(B)**. Two-tailed unpaired *t* test **(C–E)**. ns, not significant; *P < 0.05, **P < 0.01, ****P < 0.0001.

Analysis of clinical data derived from our medical center showed that therapeutic response to EGFR-TKIs, including osimertinib, in patients with advanced stage tumors (T3-T4) was worse than those with earlier stage tumors (T1-T2). Clinical response was assessed at the 8^th^ month of treatment, which shows that partial response (PR) and stable disease (SD) were more frequently observed in cases with smaller tumors (T1-T2), while progressed disease (PD) predominantly occurred to patients with more advanced tumor stages (T3-T4) (χ2 = 7.4, P=0.025) (**Figure 2A**). Emerging evidence suggests that advanced tumors usually have a more immunosuppressive TME than early-stage tumors (14). We hypothesized that advanced tumors with more immunosuppressive TME are less responsive to osimertinib. To test this hypothesis, we delayed osimertinib treatment until PE tumor size reaching 400 mm^3^. As **Figure 2B** shows, osimertinib (10mg/kg, po, qd) treatment slowed down tumor growth initially but failed to induce tumor regression, and these tumors eventually progressed through the treatment and presented growth rates comparable to control. We also found that osimertinib did not increase T cell infiltration or activation in these more advanced PE tumors (**Figure 2C**, **Supplementary Figure 2A**). To investigate this further, we compared tumor-infiltrating immune cells in small (~100 mm^3^) and large (~500 mm^3^) PE tumors without treatment. Our data revealed that T cells and dendritic cells (DCs) were significantly reduced in large tumors as compared to small tumors **(** **Figure 2D**, **Supplementary Figures 2B, C**). Notably, TAMs accounted for ~75% of CD45^+^ cells in large tumors *vs.* 22% of that in small tumors (**Figure 2E**, **Supplementary Figures 2B, C**). Moreover, M2-like TAMs (MHC II^low^, CD206^+^) were significantly increased in large PE tumors (**Figure 2E**, **Supplementary Figures 2B, C**). These results suggest that an immunosuppressive TME dominated by M2-like TAMs was developed in large PE tumors that are resistant to osimertinib.

### TAMs inactivate T cells and diminish therapeutic efficacy of osimertinib

**Figure 3:**
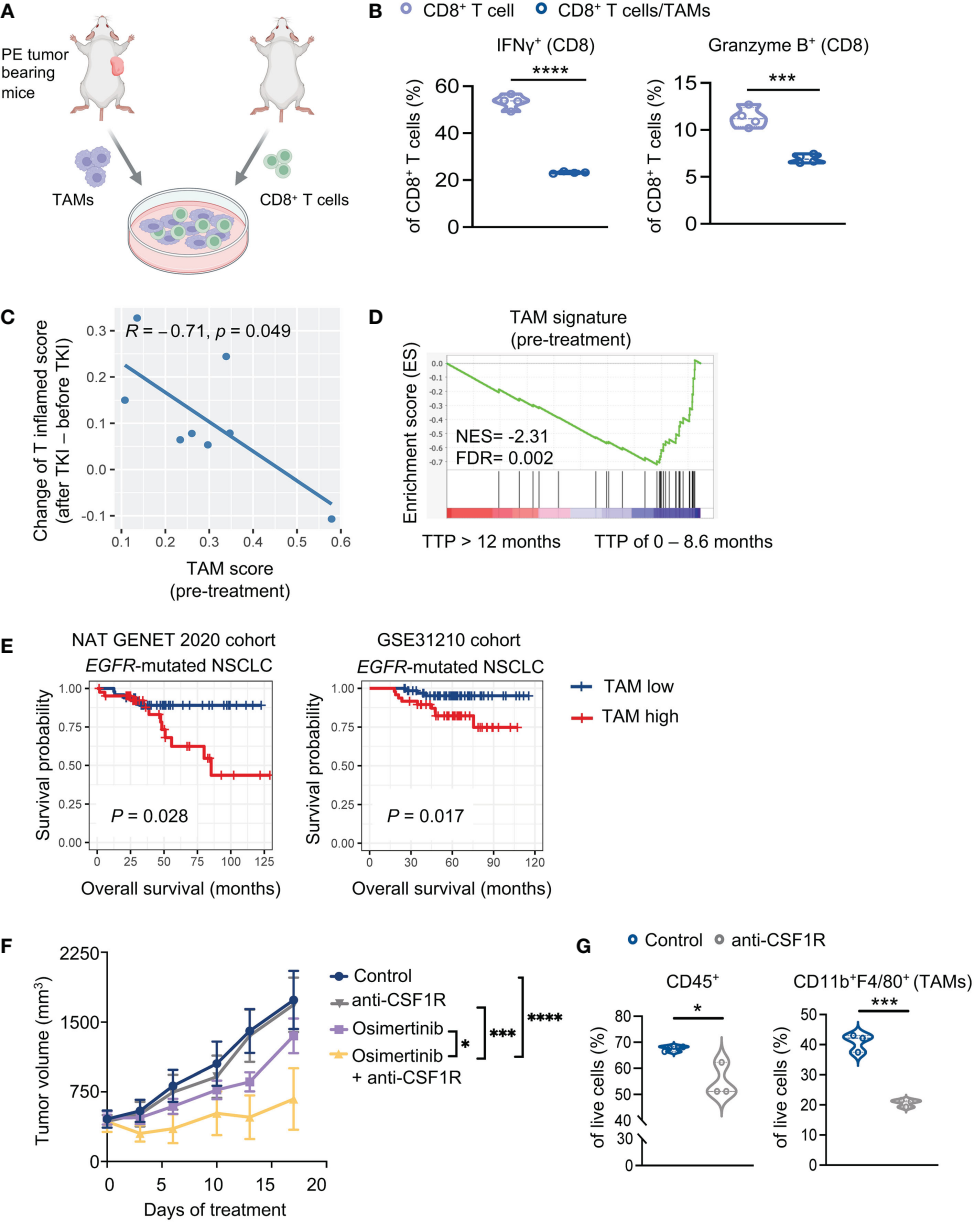
TAMs suppress T cells and diminish therapeutic efficacy of osimertinib. **(A)** A schematic diagram demonstrates the experiment of **(B)**. TAMs (7AAD^−^ CD45^+^ CD11b^+^ F4/80^+^) harvested from advanced PE tumors were co-cultured with splenic CD8^+^ T cells isolated from naïve mice. **(B)** Analysis of cytokine production by CD8^+^ T cells (CD8^+^ T cells, n = 4; CD8^+^ T cells + TAMs, n =4). **(C, D)** Analysis of a cohort of eight patients with *EGFR* mutant lung cancers who received an EGFR-TKI (osimertinib or erlotinib) as the first-line treatment. **(C)** Pearson correlation analysis of enrichment scores of TAMs (pre-treatment) with increase of T cell inflamed scores upon EGFR-TKIs. Each dot represents one patient. n = 8. **(D)** Analysis of TAM signature of patients prior to EGFR-TKI treatment. Patients were grouped according to the duration of response [TTP of 0 – 8.6 month (n = 4 patients) or >12 months (n = 4 patients)]. **(E)** Survival implication of TAM score among NSCLC patients harboring *EGFR* mutation. NSCLC patients harboring *EGFR* mutation were stratified into two subgroups according to TAM enrichment scores (high, > mean value; low, < mean value), whose overall survival (OS) were compared through Kaplan-Meier survival analysis. OS was defined as time from surgery to date of death. **Left**, Nat Genet 2020 cohort (*EGFR-*mutant NSCLC, n = 91). **Right**, GSE31210 cohort (*EGFR-*mutant NSCLC, n = 127). **(F)** Tumor growth of PE allografts in FVB mice treated with osimertinib (10 mg/kg, p.o., q.d.) and anti-CSF1R (40 mg/kg, i.p., thrice a week) as monotherapy or in combination. Treatments were started when tumors reached approximately 400 mm^3^. Control, n = 6; anti-CSF1R, n = 6; osimertinib, n = 7; osimertinib + anti-CSF1R, n = 5. **(G)** Flow cytometry analysis of PE tumors in FVB mice after 7 days of treatment with or without anti-CSF1R (40 mg/kg, i.p., thrice a week). Each dot represents data from a single tumor. Data are presented as median with quartiles (violin plots, **B, G**) or mean ± SEM **(F)**. Two-tailed unpaired *t* test **(B, G)**. Two-way ANOVA **(F)**. *P < 0.05, ***P < 0.001, ****P < 0.0001.

To evaluate immunosuppressive functions of TAMs of PE tumors, we performed an *ex vivo* co-coculture experiment with TAMs and CD8^+^ T cells. TAMs were isolated from large PE tumors (> 400 mm^3^) and co-cultured with splenic CD8^+^ T cells isolated from naïve FVB mice (**Figure 3A**). We found that PE TAMs, but not bone marrow-derived macrophages (BMDMs) from healthy control mice, inhibited IFNγ and Granzyme B production of CD8^+^ T cells (**Figure 3B**, **Supplementary Figure 3A**). We further showed that co-culturing of BMDMs with PE tumor cells potently increased expression of inducible nitric oxide synthase (iNOS) and programmed death-ligand 1 (PD-L1) in macrophages, while arginase 1 and TGF-β were not significantly altered in macrophages (**Supplementary Figure 3B**). Evidence from previous studies have shown immunosuppressive effects of PD-L1 and nitric oxide (NO) on T cells (25–28). Thus, our results suggest potential mechanisms underlying macrophages-mediated suppression of CD8^+^ T cells in PE tumors.

To assess the clinical relevance of our observation, we re-visited RNA-seq data of patients with *EGFR*-mutant lung cancers derived from tumors biopsied prior to and after EGFR-TKI (osimertinib or erlotinib) treatment (6). As shown in **Figure 3C**, the enrichment scores of TAM signature (21) before treatment were negatively correlated with T cell activation upon TKI treatment, as indicated by the increase of T cell inflamed scores. Further analysis of the same data set revealed that the TAM signature was significantly enriched in patients with short duration of response (TTP of 0 – 8.6 month) relative to patients with prolonged response (TTP > 12 months) (**Figure 3D**). These data suggest that TAMs of *EGFR*-mutant lung tumors may inhibit T cell activation elicited by EGFR-TKIs and affect therapeutic effects of the drugs. Moreover, we analyzed additional patient data of two clinical datasets from cBioPortal and Cancer Genomics and NCBI’s gene expression omnibus (GEO), i.e., NAT GENET 2020 cohort (17) and GSE31210 cohort (18). We found that patients with higher TAM abundance (as indicated by enrichment score of TAM signature) are associated with worse overall survival, and interestingly, this association is more significant in patients with *EGFR*-mutant NSCLC (**Figure 3E**, **Supplementary Figures 3C, D**).

To further investigate the role of TAMs *in vivo*, we treated large PE tumor-bearing FVB mice with an anti-CSF1-R antibody to deplete TAMs. We found that while anti-CSF1R alone had little therapeutic effects, it significantly improved the response of advanced PE tumors to osimertinib (**Figure 3F**). Flow cytometry analysis confirmed that anti-CSF1-R significantly reduced CD45^+^ cells and TAMs in large PE tumors (**Figure 3G**). Together, these data confirmed the suppressive effects of TAMs on the therapeutic efficacy of osimertinib.

### Combination of a systemic STING agonist with osimertinib induces regression of advanced PE tumors

**Figure 4:**
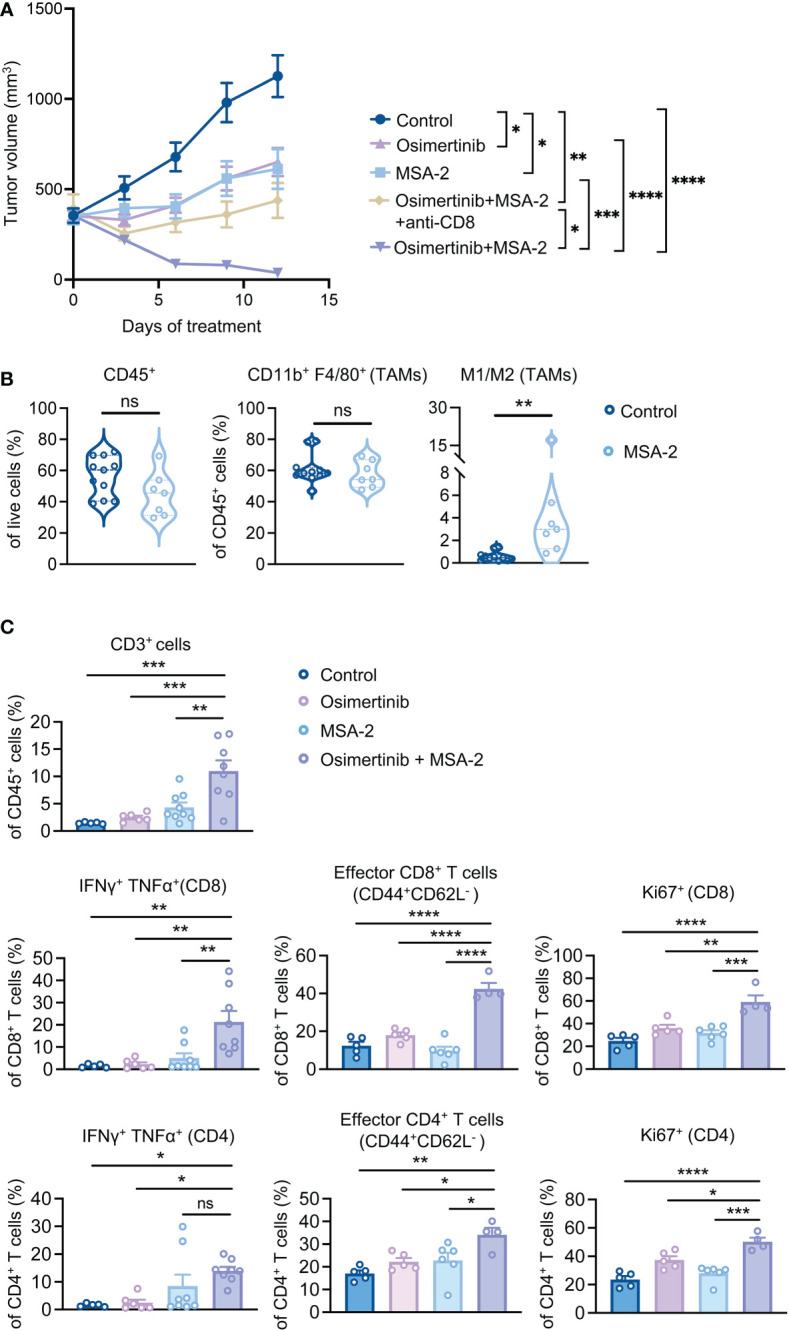
Combination of a systemic STING agonist MSA-2 with osimertinib induces regression of advanced PE tumors. **(A)** Tumor growth of PE allografts in FVB mice treated with osimertinib (10 mg/kg, p.o., q.d.) and MSA-2 (20 mg/kg, i.p., every three days) as a single agent or in combination, and with or without an anti-CD8 neutralizing antibody. Treatments were started when tumor size reached approximately 400 mm^3^. Control, n = 6; osimertinib, n = 8; MSA-2, n = 8; osimertinib + MSA-2, n = 10; osimertinib + MSA-2 + anti-CD8, n = 8. **(B, C)** Flow cytometry analysis of PE tumors in FVB mice after 2 weeks of treatments. Each dot represents data from a single tumor. **(B)** Analysis of CD45^+^ leukocytes, tumor-associated macrophages (TAMs; CD45^+^ CD11b^+^ F4/80^+^), and M1 (MHC-II^high^ CD206^−^)/M2 (MHC-II^low^ CD206^+^) ratios of TAMs. **(C)** Analysis of CD3^+^ T cell infiltration, and intratumoral CD8^+^ and CD4^+^ T cells for production of effector cytokines (IFNγ and TNFα), proportion of effector cells (CD44^high^CD62L^low^), and intracellular expression of Ki67. Data are presented as mean ± SEM **(A, C)** or median with quartiles (violin plots, **B**). Two-way ANOVA **(A)**. Two-tailed unpaired *t* test **(B)**. One-way ANOVA **(C)**. ns, not significant; *P < 0.05, **P < 0.01, ***P < 0.001, ****P < 0.0001.

We have recently shown that reprogramming TAMs with a STING agonist exerts superior therapeutic effects than TAM depletion in breast cancer (29). To investigate TAM reprogramming strategy in advanced PE tumors, we employed MSA-2, a small molecule non-nucleotide STING agonist suitable for systemic administration (30). We first tested whether reprogramming TAMs with a STING agonist can reverse the immunosuppressive effects of TAMs on CD8^+^ T cells, we pre-treated TAMs isolated from large PE tumors (> 400 mm^3^) with MSA-2 before co-culturing with CD8^+^ T cells. Indeed, TAMs pre-treated with MSA-2 enabled CD8^+^ T cell activation as evidenced by the increased production of IFNγ, Granzyme B, and TNFα (**Supplementary Figure 4A**). To evaluate TAM reprogramming strategy *in vivo*, PE tumor-bearing FVB mice were subjected to osimertinib and MSA-2 as monotherapy or in combination when tumor size reached about 400 mm^3^ (**Figure 4A**). Strikingly, while osimertinib or MSA-2 monotherapy only slowed down tumor growth, the combination resulted in tumor regression (**Figure 4A**). Moreover, depletion of CD8^+^ T cells significantly compromised the efficacy of the combination therapy (**Figure 4A**), suggesting the contribution of adaptive immunity to the tumor regression elicited by osimertinib + MSA-2. Immune profiling of PE tumors showed that MSA-2 promoted anti-tumor polarization of TAMs, as evidenced by significantly increased M1/M2 ratio, without changing TAM abundance in CD45^+^ cells (**Figure 4B**, **Supplementary Figure 4B**). Moreover, combined MSA-2 with osimertinib increased intratumor T cell infiltration, up-regulated production of anti-tumor cytokines in both CD8^+^ and CD4^+^ T cells (i.e., IFNγ and TNFα), increased intratumoral effector CD8^+^ and CD4^+^ T cells (CD44^high^CD62L^low^), as well as enhanced the proliferation of both CD8^+^ and CD4^+^ T cells as evidenced by the increase of Ki67^+^ cells (**Figure 4C**, **Supplementary Figure 4C**).

Many solid tumors have been reported to become STING deficient during tumor development and progression (31–33). Of note, we found that STING protein level is dramatically reduced in PE tumor cells compared to TAMs isolated from PE tumors (**Supplementary Figure 4D**). Consistently, MSA-2 treatment triggered IFNβ production in TAMs but not in PE tumor cells (**Supplementary Figure 4E**). These data suggest that the contribution of the STING pathway activation in tumor cells is less significant than that of macrophages to the response of PE tumors to MSA-2. Together, our data demonstrate that combined osimertinib with MSA-2 reprogrammed immunosuppressive TME and led to tumor regression of large and more aggressive PE tumors.

## Discussion

There is a lack of development in combining EGFR-TKIs with immunotherapies for the treatment of patients with advanced *EGFR*-mutant NSCLC. Of note, how the interactions between tumor cells and immune system affect the response to EGFR-TKI therapy is not completely understood, which limits the development of an effective combination therapy. Here, we report a previously undefined role of TAMs in impeding the efficacy of osimertinib, a third generation EGFR-TKI. Notably, reprogramming TAMs with a STING agonist, rather than depletion of TAMs, synergized with osimertinib in inducing regression of advanced tumors in mice. These findings provide a preclinical proof of concept that combining osimertinib with a TAM-targeting agent could improve the response of advanced tumors to osimertinib.

Evidence from recent studies has suggested the involvement of immune response in the antitumor activity of EGFR-TKI (6, 34, 35). In agreement with these findings, we found that the therapeutic efficacy of osimertinib was significantly compromised when CD8^+^ T cells were depleted. The mechanism underlying osimertinib-elicited T cell activation is still unfolding. One explanation is that this is a consequence of inflammatory cell death induced by osimertinib (36). The other explanation is that osimertinib reverses immunosuppressive functions of oncogenic EGFR mutations. Sugiyama et al. show that oncogenic activation of EGFR increases CCL22-mediated recruitment of regulatory T cells *via* activation of cJun/cJun N-terminal kinase, and decreases the production of CD8^+^ T cell recruiting chemokines including CXCL10 and CCL5 *via* suppressing interferon regulatory factor-1 (34). Another study by Fang et al. also suggest that EGFR activation inhibits interferon pathway in tumor cells (35).

Macrophages have been previously shown to contribute to the progression of *EGFR*-mutant lung cancer (37). In this study, we demonstrated the role of TAMs in limiting the efficacy of osimertinib. The mechanism underlying TAM-mediated resistance to osimertinib is likely through its suppressive function on T cells, which is important for the antitumor activity of osimertinib. Macrophages have been reported as a major immunosuppressive component that blocks T cell functions in TME (7). Mechanistically, our data suggest that TAMs of *EGFR*-mutant lung tumors may suppress T cells through expression of nitric oxide synthase and PD-L1. In agreement with our findings, recent studies have shown a strong correlation of PD-L1 expression on tumor-infiltrating immune cells, including TAMs, with immunosuppression and tumor aggressiveness in non-small cell lung cancer (25, 26). On the other hand, nitric oxide (NO) generated by iNOS of M2-polarized TAMs has been shown to contribute to the resistance of lung cancer cells to cisplatin (38). NO has also been reported to suppress T cell proliferation (27, 28). In addition to suppressing T cells, TAMs were also reported to mediate resistance to chemotherapy and other anti-tumor treatments through providing survival factors to and/or activating anti-apoptotic programs in malignant cells (39–41). While our current study highlights the immunosuppressive role of TAMs, further studies should address whether other TAM-mediated mechanisms are involved in EGFR-TKI resistance.

We have shown that exogenous STING agonist can efficiently reprogram TAM phenotype from a pro-tumorigenic to an anti-tumorigenic state, which may stimulate T cell cross-priming and trigger a robust adaptive antitumor immunity (29). In the present study, the combination of osimertinib with a novel systemic STING agonist MSA-2 induced regression of advanced *EGFR*-mutant tumors in mice. The requirement of T cells is evidenced by the finding that therapeutic efficacy was significantly compromised in the absence of CD8^+^ T cells. In addition to M1-like TAMs, we do not exclude the contributions of other cell types to the efficacy of the combination therapy, e.g. DCs and endothelial cells, which can also be responders to a STING agonist in eliciting anti-tumor immunity (42, 43).

Our data suggest a role of TME in the response of *EGFR*-mutant NSCLC to osimertinib. However, the mechanism(s) contributing to the formation of a TAM-enriched TME in *EGFR*-mutant NSCLC remains unanswered. Investigating the complex and dynamic interactions between tumor cells and immune cells and their effects on disease progression and therapeutic response is an active field of immune-oncology research. Another limitation of the current study is the small sample size of the clinical cohorts that are available to us. Further validation with larger patient cohorts is needed in future studies. While MSA-2 is a next-generation small molecule STING agonist that can be administrated systemically (30), future studies evaluating the combination of an EGFR-TKI with a clinically tested STING agonist would accelerate clinical translation. Nevertheless, our study highlights the potential of a rational combination strategy to improve the therapeutic efficacy of osimertinib in *EGFR* mutant-driven lung cancers.

## Data availability statement

The datasets analyzed for this study can be found in the Gene Expression Omnibus (GEO) repository (https://identifiers.org/geo:GSE165019) or cBioPortal (http://www.cbioportal.org).

## Ethics statement

The studies involving human participants were reviewed and approved by Ethics Committee of First Affiliated Hospital of Sun Yat-sen University. The patients/participants provided their written informed consent to participate in this study.

The animal study was reviewed and approved by DFCI Institutional Animal Care and Use Committee (IACUC).

## Author contributions

QW and JZ conceived the project. ZL and QW performed most of experiments. TJ and WW provided assistance for animal experiments. ZL, QW, and ZJ analyzed the results and wrote the manuscript. All authors contributed to the article and approved the submitted version.

## Supplementary material

Supplementary Figure 1 Osimertinib-elicited activation of T cells is important for the anti-tumor efficacy of osimertinib *in vivo.* **(A)** Western blots of PE tumor cells treated with osimertinib (300 nM or 600 nM) or erlotinib (300 nM or 600 nM) for 6 hours. **(B)** IC_50_ (fold change) of PE tumor cells treated with osimertinib or erlotinib for 3 days. **(C)** Flow cytometry analysis of PE tumors and tumor drainage lymph nodes (TDLNs) in FVB mice following 11 days of treatments with osimertinib (10 mg/kg, p.o., q.d.) or osimertinib + anti-CD8 (400 μg/mouse, i.p.). Treatment was started when tumor volume reached approximately 60 mm^3^. **(D, E)** Gating strategies **(D)** and representative plots **(E)** of flow cytometry analysis in **Figure 1D**. Data are presented as mean ± SEM **(B)** or median with quartiles (violin plots, **C**). Two-tailed unpaired *t* test **(B, C)**. ns, not significant; *****P* < 0.0001.

Supplementary Figure 2 Advanced PE tumors dominated by M2-like TAMs are resistant to osimertinib. **(A)** Representative plots of flow cytometry analysis in **Figure 2C**. **(B, C)** Gating strategies **(B)** and representative plots **(C)** of flow cytometry analysis in **Figure 2D, E**.

Supplementary Figure 3 TAMs suppress T cells and diminish therapeutic efficacy of osimertinib. **(A)** Flow cytometry analysis of cytokine production by CD8^+^ T cells co-cultured with or without bone marrow-derived macrophages (BMDMs) from naïve mice (CD8^+^ T cells, n = 4; CD8^+^ T cells/BMDMs, n =4). **(B)** Flow cytometry analysis of control bone marrow-derived macrophages (BMDMs) and PE tumor-educated macrophages (TEMs). TEMs were derived by co-culturing BMDMs with PE tumor cells at a ratio of 1:1 for one day (BMDMs, n = 4; TEMs, n =4). **(C, D)** NSCLC patients regardless of *EGFR* mutation status were stratified into two subgroups according to TAM enrichment scores (high, > mean value; low, < mean value), whose overall survival (OS) were compared through Kaplan-Meier survival analysis. OS was defined as time from surgery to date of death. **(C)** Analysis of Nat Genet 2020 cohort (n = 305). **(D)** Analysis GSE31210 cohort (n = 226). Data are presented as median with quartiles (violin plots, **A, B**). Two-tailed unpaired *t* test **(A**, **B)**. ns, not significant; ****P* < 0.001.

Supplementary Figure 4 Combination of a systemic STING agonist MSA-2 with osimertinib induces regression of advanced PE tumors. **(A)** Flow cytometry analysis of cytokine production by CD8^+^ T cells co-cultured with PE TAMs pre-treated with or without MSA-2 (33 μM, 1 day) for 2 days (CD8^+^ T/TAM, n = 4; CD8^+^ T/MSA-2 pretreated TAM, n = 4). **(B)** Representative plots of flow cytometry analysis in **Figure 4B**. **(C)** Gating strategies of flow cytometry analysis in **Figure 4C**. **(D)** Western blots for STING and VINCULIN in PE tumor cells and PE TAMs. **(E)** ELISA analysis of IFNβ in media from PE tumor cells and PE TAMs with or without one day of MSA-2 (33 μM) treatment. Data are presented as median with quartiles (violin plots, **A**) or mean ± SEM **(E)**. Two-tailed unpaired *t* test **(A)**. One-way ANOVA **(E)**. ns, not significant; ** *P* < 0.01, *****P* < 0.0001.

## References

[1] de Groot PM Wu CC Carter BW Munden RF. The epidemiology of lung cancer. Transl Lung Cancer Res (2018) 7:220–33. doi: 10.21037/tlcr.2018.05.06 PMC6037963 30050761

[2] Rosell R Moran T Queralt C Porta R Cardenal F Camps C. Screening for epidermal growth factor receptor mutations in lung cancer. N Engl J Med (2009) 361:958–67. doi: 10.1056/NEJMoa0904554 19692684

[3] Shi Y Au JS Thongprasert S Srinivasan S Tsai CM Khoa MT. A prospective, molecular epidemiology study of EGFR mutations in Asian patients with advanced non-small-cell lung cancer of adenocarcinoma histology (PIONEER). J Thorac Oncol (2014) 9:154–62. doi: 10.1097/JTO.0000000000000033 PMC4132036 24419411

[4] Ramalingam SS Vansteenkiste J Planchard D Cho BC Gray JE Ohe Y. Overall survival with osimertinib in untreated, EGFR-mutated advanced NSCLC. N Engl J Med (2020) 382:41–50. doi: 10.1056/NEJMoa1913662 31751012

[5] Leonetti A Sharma S Minari R Perego P Giovannetti E Tiseo M. Resistance mechanisms to osimertinib in EGFR-mutated non-small cell lung cancer. Br J Cancer (2019) 121:725–37. doi: 10.1038/s41416-019-0573-8 PMC6889286 31564718

[6] Gurule NJ McCoach CE Hinz TK Merrick DT Van Bokhoven A Kim J. A tyrosine kinase inhibitor-induced interferon response positively associates with clinical response in EGFR-mutant lung cancer. NPJ Precis Oncol (2021) 5:41. doi: 10.1038/s41698-021-00181-4 34001994 PMC8129124

[7] Ruffell B Chang-Strachan D Chan V Rosenbusch A Ho CM Pryer N. Macrophage IL-10 blocks CD8+ T cell-dependent responses to chemotherapy by suppressing IL-12 expression in intratumoral dendritic cells. Cancer Cell (2014) 26:623–37. doi: 10.1016/j.ccell.2014.09.006 PMC4254570 25446896

[8] Salvagno C Ciampricotti M Tuit S Hau CS van Weverwijk A Coffelt SB. Therapeutic targeting of macrophages enhances chemotherapy efficacy by unleashing type I interferon response. Nat Cell Biol (2019) 21:511–21. doi: 10.1038/s41556-019-0298-1 PMC6451630 30886344

[9] Ruffell B Coussens LM. Macrophages and therapeutic resistance in cancer. Cancer Cell (2015) 27:462–72. doi: 10.1016/j.ccell.2015.02.015 PMC4400235 25858805

[10] Zhang Y Fu F Hu H Wang S Li Y Hu H. Gefitinib as neoadjuvant therapy for resectable stage II-IIIA non-small cell lung cancer: A phase II study. J Thorac Cardiovasc Surg (2021) 161:434–442.e2. doi: 10.1016/j.jtcvs.2020.02.131 32340810

[11] Gray JE Okamoto I Sriuranpong V Vansteenkiste J Imamura F Lee JS. Tissue and plasma EGFR mutation analysis in the FLAURA trial: Osimertinib versus comparator EGFR tyrosine kinase inhibitor as first-line treatment in patients with EGFR-mutated advanced non-small cell lung cancer. Clin Cancer Res an Off J Am Assoc Cancer Res (2019) 25:6644–52. doi: 10.1158/1078-0432.CCR-19-1126 PMC7209579 31439584

[12] Ramalingam SS Yang JC Lee CK Kurata T Kim DW John T. Osimertinib as first-line treatment of EGFR mutation-positive advanced non-Small-Cell lung cancer. J Clin Oncol (2018) 36:841–9. doi: 10.1200/JCO.2017.74.7576 28841389

[13] Rizvi NA Rusch V Pao W Chaft JE Ladanyi M Miller VA. Molecular characteristics predict clinical outcomes: prospective trial correlating response to the EGFR tyrosine kinase inhibitor gefitinib with the presence of sensitizing mutations in the tyrosine binding domain of the EGFR gene. Clin Cancer Res an Off J Am Assoc Cancer Res (2011) 17:3500–6. doi: 10.1158/1078-0432.CCR-10-2102 PMC3261615 21558399

[14] Kim SI Cassella CR Byrne KT. Tumor burden and immunotherapy: Impact on immune infiltration and therapeutic outcomes. Front Immunol (2020) 11:629722. doi: 10.3389/fimmu.2020.629722 33597954 PMC7882695

[15] Weischenfeldt J Porse B. Bone marrow-derived macrophages (BMM): Isolation and applications. CSH Protoc (2008) 2008:pdb prot5080. doi: 10.1101/pdb.prot5080 21356739

[16] Guo X Zhou Y Wu T Zhu X Lai W Wu L. Generation of mouse and human dendritic cells in vitro. J Immunol Methods (2016) 432:24–9. doi: 10.1016/j.jim.2016.02.011 26876301

[17] Chen J Yang H Teo ASM Amer LB Sherbaf FG Tan CQ. Genomic landscape of lung adenocarcinoma in East asians. Nat Genet (2020) 52:177–86. doi: 10.1038/s41588-019-0569-6 32015526

[18] Okayama H Kohno T Ishii Y Shimada Y Shiraishi K Iwakawa R. Identification of genes upregulated in ALK-positive and EGFR/KRAS/ALK-negative lung adenocarcinomas. Cancer Res (2012) 72:100–11. doi: 10.1158/0008-5472.CAN-11-1403 22080568

[19] Subramanian A Tamayo P Mootha VK Mukherjee S Ebert BL Gillette MA. Gene set enrichment analysis: a knowledge-based approach for interpreting genome-wide expression profiles. Proc Natl Acad Sci USA (2005) 102:15545–50. doi: 10.1073/pnas.0506580102 PMC1239896 16199517

[20] Love MI Huber W Anders S. Moderated estimation of fold change and dispersion for RNA-seq data with DESeq2. Genome Biol (2014) 15:550. doi: 10.1186/s13059-014-0550-8 25516281 PMC4302049

[21] Cassetta L Fragkogianni S Sims AH Swierczak A Forrester LM Zhang H. Human tumor-associated macrophage and monocyte transcriptional landscapes reveal cancer-specific reprogramming, biomarkers, and therapeutic targets. Cancer Cell (2019) 35:588–602.e10. doi: 10.1016/j.ccell.2019.02.009 30930117 PMC6472943

[22] Ayers M Lunceford J Nebozhyn M Murphy E Loboda A Kaufman DR. IFN-γ-related mRNA profile predicts clinical response to PD-1 blockade. J Clin Invest (2017) 127:2930–40. doi: 10.1172/JCI91190 PMC5531419 28650338

[23] Eisenhauer EA Therasse P Bogaerts J Schwartz LH Sargent D Ford R. New response evaluation criteria in solid tumours: Revised RECIST guideline (version 1.1). Eur J Cancer (2009) 45:228–47. doi: 10.1016/j.ejca.2008.10.026 19097774

[24] Goldstraw P Chansky K Crowley J Rami-Porta R Asamura H Eberhardt WE. The IASLC lung cancer staging project: Proposals for revision of the TNM stage groupings in the forthcoming (Eighth) edition of the TNM classification for lung cancer. J Thorac Oncol (2016) 11:39–51. doi: 10.1016/j.jtho.2015.09.009 26762738

[25] Sumitomo R Hirai T Fujita M Murakami H Otake Y Huang CL. PD-L1 expression on tumor-infiltrating immune cells is highly associated with M2 TAM and aggressive malignant potential in patients with resected non-small cell lung cancer. Lung Cancer (2019) 136:136–44. doi: 10.1016/j.lungcan.2019.08.023 31499335

[26] Shinchi Y Ishizuka S Komohara Y Matsubara E Mito R Pan C. The expression of PD-1 ligand 1 on macrophages and its clinical impacts and mechanisms in lung adenocarcinoma. Cancer Immunol Immunother (2022) 71:2645–61. doi: 10.1007/s00262-022-03187-4 PMC8963674 35352168

[27] Sato K Ozaki K Oh I Meguro A Hatanaka K Nagai T. Nitric oxide plays a critical role in suppression of T-cell proliferation by mesenchymal stem cells. Blood (2007) 109:228–34. doi: 10.1182/blood-2006-02-002246 16985180

[28] Navasardyan I Bonavida B. Regulation of T cells in cancer by nitric oxide. Cells 10 (2021) 10(10):2655. doi: 10.3390/cells10102655 PMC8534057 34685635

[29] Wang Q Bergholz JS Ding L Lin Z Kabraji SK Hughes ME. STING agonism reprograms tumor-associated macrophages and overcomes resistance to PARP inhibition in BRCA1-deficient models of breast cancer. Nat Commun (2022) 13:3022. doi: 10.1038/s41467-022-30568-1 35641483 PMC9156717

[30] Pan BS Perera SA Piesvaux JA Presland JP Schroeder GK Cumming JN. An orally available non-nucleotide STING agonist with antitumor activity. Sci 369 (2020) 369(6506):eaba6098. doi: 10.1126/science.aba6098 32820094

[31] Konno H Yamauchi S Berglund A Putney RM Mule JJ Barber GN. Suppression of STING signaling through epigenetic silencing and missense mutation impedes DNA damage mediated cytokine production. Oncogene (2018) 37:2037–51. doi: 10.1038/s41388-017-0120-0 PMC6029885 29367762

[32] Xia T Konno H Ahn J Barber GN. Deregulation of STING signaling in colorectal carcinoma constrains DNA damage responses and correlates with tumorigenesis. Cell Rep (2016) 14:282–97. doi: 10.1016/j.celrep.2015.12.029 PMC4845097 26748708

[33] de Queiroz N Xia T Konno H Barber GN. Ovarian cancer cells commonly exhibit defective STING signaling which affects sensitivity to viral oncolysis. Mol Cancer Res (2019) 17:974–86. doi: 10.1158/1541-7786.MCR-18-0504 PMC6445711 30587523

[34] Sugiyama E Togashi Y Takeuchi Y Shinya S Tada Y Kataoka K. Blockade of EGFR improves responsiveness to PD-1 blockade in EGFR-mutated non-small cell lung cancer. Sci Immunol 5 (2020) 5(43):eaav3937. doi: 10.1126/sciimmunol.aav3937 32005679

[35] Fang Y Wang Y Zeng D Zhi S Shu T Huang N. Comprehensive analyses reveal TKI-induced remodeling of the tumor immune microenvironment in EGFR/ALK-positive non-small-cell lung cancer. Oncoimmunology (2021) 10:1951019. doi: 10.1080/2162402X.2021.1951019 34345533 PMC8288040

[36] Rosenbaum SR Wilski NA Aplin AE. Fueling the fire: Inflammatory forms of cell death and implications for cancer immunotherapy. Cancer Discovery (2021) 11:266–81. doi: 10.1158/2159-8290.CD-20-0805 PMC7858229 33451983

[37] Wang DH Lee HS Yoon D Berry G Wheeler TM Sugarbaker DJ. Progression of EGFR-mutant lung adenocarcinoma is driven by alveolar macrophages. Clin Cancer Res an Off J Am Assoc Cancer Res (2017) 23:778–88. doi: 10.1158/1078-0432.CCR-15-2597 27496865

[38] Perrotta C Cervia D Di Renzo I Moscheni C Bassi MT Campana L. Nitric oxide generated by tumor-associated macrophages is responsible for cancer resistance to cisplatin and correlated with syntaxin 4 and acid sphingomyelinase inhibition. Front Immunol (2018) 9:1186. doi: 10.3389/fimmu.2018.01186 29896202 PMC5987706

[39] Castells M Thibault B Delord JP Couderc B. Implication of tumor microenvironment in chemoresistance: tumor-associated stromal cells protect tumor cells from cell death. Int J Mol Sci (2012) 13:9545–71. doi: 10.3390/ijms13089545 PMC3431813 22949815

[40] Correia AL Bissell MJ. The tumor microenvironment is a dominant force in multidrug resistance. Drug Resist Update (2012) 15:39–49. doi: 10.1016/j.drup.2012.01.006 PMC3658318 22335920

[41] Meads MB Gatenby RA Dalton WS. Environment-mediated drug resistance: a major contributor to minimal residual disease. Nat Rev Cancer (2009) 9:665–74. doi: 10.1038/nrc2714 19693095

[42] Ding L Kim HJ Wang Q Kearns M Jiang T Ohlson CE. PARP inhibition elicits STING-dependent antitumor immunity in Brca1-deficient ovarian cancer. Cell Rep (2018) 25:2972–2980.e5. doi: 10.1016/j.celrep.2018.11.054 30540933 PMC6366450

[43] Yang H Lee WS Kong SJ Kim CG Kim JH Chang SK. STING activation reprograms tumor vasculatures and synergizes with VEGFR2 blockade. J Clin Invest (2019) 129:4350–64. doi: 10.1172/JCI125413 PMC6763266 31343989

